# Practical Points in the Use of X-Ray and High-Frequency Currents

**Published:** 1909-12

**Authors:** 


**Practical Points in the Use of X-ray and High-Frequency Currents,** by **Aspinwall Judd,** M.D., formerly Radiologist at the Post-Graduate Medical School and Hospital, New York. New York: Rebman Co. 1909. (Cloth, $1.50.)

The increase of *x*-ray work is such as to give a book of this kind great importance and it should be in the hands of everyone who owns an electrical outfit. It not only tells all about the machine but also tells how to take care of it. It contains 180 pages of good, solid, concise, reading matter, printed in good type. The book is moderate in price. There is a need for this work which is of special importance to amateur operators of *x*-ray machines.

